# Ultra-processed foods consumption and diet quality during shift work among healthcare workers: a within-subject design

**DOI:** 10.1186/s12889-026-28697-z

**Published:** 2026-07-29

**Authors:** Azad Ilhan, Muhammet Faruk Yiğit, Yücel Büyükdere

**Affiliations:** 1 Faculty of Health Sciences, Department of Nutrition and Dietetics, Van Yüzüncü Yıl University, Van, Tuşba, 65080 Türkiye; 2 Faculty of Health Sciences, Department of Nursing, Van Yüzüncü Yıl University, Van, Türkiye; 3 Kulu Faculty of Health Sciences, Department of Nutrition and Dietetics, Selçuk University, Konya, Türkiye

**Keywords:** Shift work, Ultra-processed foods, Diet quality, Healthcare workers

## Abstract

**Background:**

While ultra-processed foods (UPF) consumption is a key determinant of metabolic health, within-subject evidence evaluating shifts in UPF intake is limited. This study compared UPF consumption and dietary quality between shift and non-shift days among healthcare workers.

**Methods:**

This within-subject observational study included 60 healthcare workers with rotating shift schedules. Four 24-hour dietary recalls (two shift days and two non-shift days) were obtained by a trained dietitian. Foods were classified by the NOVA system, and the UPF energy contribution percentage (UPFECP) was calculated. Diet quality was assessed using the Diet Quality Index (DQI). Nutrient intakes and meal patterns on shift and non-shift days were compared using paired statistical analyses. Additionally, hierarchical linear regression models were used to examine the association between condition-specific UPFECP and DQI total scores after adjustment for age, sex, body mass index, and prior nutrition education.

**Results:**

Compared with non-shift days, shift days showed lower daily energy intake (2624.8 ± 816.5 vs. 2826.6 ± 857.8 kcal, *p* = 0.025). On shift days, the energy contribution of unprocessed/minimally processed foods decreased (from 48.5% to 42.4%, *p* < 0.001), whereas UPF increased (from 8.6% to 11.8%, *p* = 0.002). While the overall frequency of meal skipping did not differ significantly between conditions (*p* = 0.261), the specific meals skipped showed a distinct shift (*p* < 0.001). Higher UPF consumption during the shift was associated with lower DQI total and DQI adequacy scores (*p* = 0.003 and *p* < 0.001, respectively). In adjusted hierarchical regression analyses, UPFECP was independently and inversely associated with DQI total score on shift days (B= -0.411, 95% CI: -0.660 to -0.161, *p* = 0.002), whereas no significant association was observed on non-shift days.

**Conclusion:**

Shift work was associated with increased UPF consumption and poorer diet quality despite lower total daily energy intake. These findings suggest that shift-related working conditions may promote unfavorable dietary patterns characterized by a greater reliance on UPF. Improving access to unprocessed/minimally processed food options during night shifts may represent a practical strategy to support the nutritional health of healthcare workers.

**Supplementary Information:**

The online version contains supplementary material available at https://doi.org/10.1186/s12889-026-28697-z.

## Background

 Ultra-processed foods (UPF) account for a significant share of daily energy intake in many high-income countries, and consumption is also rising rapidly in low- and middle-income nations [1]. Factors such as industrialization, technological advancements, changes in socioeconomic conditions, globalization, and increased fast-food consumption contribute to this trend in both developed and developing countries [2–4]. UPF are linked to higher energy intake, increased carbohydrate consumption, and a greater risk of obesity, cardiovascular disease, type 2 diabetes, cancer, metabolic disorders, and other non-communicable diseases [1, 2].

Maintaining an optimal nutritional status and a balanced diet is paramount for healthcare professionals to effectively execute their clinical duties, particularly within demanding shift-work schedules that compromise cognitive and psychological resilience. Shift work can also disrupt eating habits, affecting nutrition and health [5, 6]. Healthcare workers on rotating shifts have irregular meal schedules, increased meal frequency, and rely on snacks for energy at night [7]. Furthermore, these workers consume different food groups on shift days compared to non-shift days [8]. Healthcare professionals serve as an ideal model for studying shift work due to their unique combination of extreme circadian disruption and high health literacy. Consequently, findings from this cohort can provide transferable insights applicable to other rotating shift-work industries, such as manufacturing, transport, or hospitality.

However, the hospital food environment often fails to support healthy food choices. In particular, it leads to a preference for energy-dense, ultra-processed snacks, especially those available through vending machines and other easily accessible outlets [9]. Together, these factors increase the risk of poor diet quality among healthcare professionals who work shifts. Indeed, emerging studies among healthcare professionals underscore that rotational shift schedules and night duties often compromise overall diet quality scores, shifting nutritional behavior toward lower-quality dietary patterns [10]. Investigating this population not only captures within-subject variations that remain highly limited in field-based data, but also provides transferable insights and a behavioral model applicable to other rotating shift-work industries. To comprehensively assess these complex dietary shifts, standardized, multidimensional metrics are required. In this study, we utilized the Diet Quality Index (DQI) and the NOVA classification. The DQI is highly effective for capturing holistic nutritional adequacy and moderation across overall dietary patterns [11, 12]. Conversely, the NOVA classification provides a validated framework for quantifying the degree of food processing—a critical factor in hospital vending environments [13]. Together, these complementary metrics overcome individual limitations to provide a robust dual assessment of nutrient profiling and processing patterns.

Although studies on shift work and nutrition are increasing, most use cross-sectional designs, which limit control for personal and between-person differences. Within-subject approaches study the same people under the same work conditions. This can better detect shift-related dietary changes by reducing time-invariant confounding. Still, little is known about how UPF consumption differs between shift and non-shift work days among healthcare professionals with rotating schedules. Given the growing recognition of UPF as an important determinant of diet quality and long-term metabolic health, understanding its contribution to daily energy intake during shift work may provide insights into the nutritional burden associated with irregular working schedules. Therefore, this study aims to more comprehensively assess the effects of shift work on diet quality by comparing the UPF energy contribution percentage (UPFECP) on both shift and non-shift days.

## Methods

This was a within-subject repeated-measures study in which measurements were taken from each participant on separate days. The study population consisted of Turkish healthcare workers who work shifts, who were recruited using a convenience sampling approach based on voluntary participation. To be eligible for inclusion, participants were required to have been actively working under this rotating shift schedule for at least 6 months before data collection to ensure adaptation to the work environment. All participants worked a total of 40 h per week. The work schedule was classified into two distinct conditions: a non-shift day, which consisted of a standard 8-hour daytime from 08:00 to 16:00, and a shift day, which involved 16 h spanning from 16:00 to 08:00. The sample size was calculated with the G*Power software (version 3.1.9.7). To determine the sample size, a power analysis was conducted using the paired samples t-test for the dependent variables. In the G*Power program, the parameters were set to an effect size of 0.50, a Type I error rate (α) of 0.05, and a power (1-β) of 0.95. The calculation resulted in a sample size of 60 participants. Pregnant and breastfeeding women, as well as individuals with a mental health condition (self-reported), were excluded from the study.

A researcher-developed questionnaire included ‘General Information’ and ‘Food Consumption Record’ sections. The full English version of the survey questionnaire is provided in supplementary file. It was administered to shift-working healthcare workers who agreed to participate in the study. Anthropometric parameters, including body weight (kg) and height (cm), were measured by a trained dietitian using an electronic scale and a wall-mounted stadiometer, with participants wearing light clothing and barefoot, aligned in the Frankfort plane. BMI (kg/m^2^) was calculated as weight/height^2^.

### Dietary intake assessment

A 24-hour dietary recall for four days (two shift and two non-shift days) was obtained by a trained dietitian via face-to-face interview. While 24-hour dietary recalls are inherently susceptible to recall bias, this limitation was minimized by conducting interviews using a structured five-step multiple-pass protocol with targeted probes designed to capture commonly forgotten items, such as nocturnal snacks and workplace beverages. Dietary recalls for both shift and non-shift days were collected at two-week (10–14-day) intervals, following the European Food Safety Authority (EFSA) recommendation [14]. To minimize potential seasonal variations in dietary patterns, all data collection was strictly conducted within a homogeneous two-month summer period (July and August). The ‘Food and Nutrition Photo Catalog: Measurements and Quantities’ was utilized to estimate the quantities of foods and beverages consumed [15]. ‘Standardized Recipes’ were applied to determine the amounts of foods included in the meals [16]. Daily energy and nutrient intakes were calculated using the Nutrition Information System (BeBiS) version 8.1. Dietary data were audited for implausible energy intakes using clinical cutoffs (< 500 kcal/day or > 5000 kcal/day). No participants fell outside these criteria, confirming the validity of the reported intakes. To assess the adequacy of daily energy and nutrient intake, the Turkish Recommended Dietary Allowance (RDA) was used according to age and gender [17]. Nutrient intake below two-thirds of the RDA (< 67%) was considered low. Additionally, meal skipping was categorized as the self-reported omission of a major baseline meal (breakfast or lunch) during its culturally established daily time window.

### Assessment of the consumption of ultra-processed foods

The NOVA classification was used to determine the processed food content of participants’ diets. Foods were classified into four groups based on FAO recommendations: unprocessed/minimally processed foods, processed ingredients, processed foods, and UPF [13]. The UPFECP was calculated by dividing the daily energy intake from UPF by the daily energy intake from all foods, then multiplying by 100.

### Diet quality

Diet quality was assessed using the International Diet Quality Index (DQI) developed by Kim and colleagues [10], which evaluates different aspects of the diet through sections with varying weighting ratios. The DQI comprises four sections: variety (0–20 points), adequacy (0–40 points), moderation (0–30 points), and overall dietary balance (0–10 points). These sections are summed to yield a DQI total score of 0 to 100, with higher scores indicating better diet quality. The index was constructed using specific criteria across its domains: the variety and adequacy components were scored based on the absolute values of daily food group portions (servings/day), nutrient weights (g/day), and the percentages of compliance with individual national guidelines. In contrast, the moderation and overall dietary balance components were calculated using the percentage of total daily energy contributed by specific macronutrients, fatty acid ratios, and absolute milligram (mg/day) values for sodium and cholesterol.

### Statistical analysis

All statistical analyses were performed using IBM SPSS Statistics version 27. To assess the data’s conformity to a normal distribution, visual methods (histogram, Q-Q plot) and statistical tests (Kolmogorov–Smirnov/Shapiro–Wilk) were applied. Depending on the variable characteristics and normality assumptions, the paired-samples t-test, one-way ANOVA, and McNemar test were utilized for group comparisons. For all analyses, a p-value of < 0.05 was considered statistically significant. To identify group differences following significant findings, the Bonferroni test was used as a post hoc analysis.

### Hierarchical linear regression models

Hierarchical multiple linear regression analyses using the enter method were conducted separately for non-shift and shift days to examine whether the condition-specific percentage of daily energy intake derived from UPF, defined as the ultra-processed food energy contribution percentage (UPFECP), was associated with DQI total score after adjustment for relevant covariates. Covariates were selected a priori based on their theoretical and methodological relevance to both UPF consumption and diet quality. Age and sex were included as key demographic factors that may influence dietary behaviors and nutrient intake. BMI was included as an anthropometric indicator potentially associated with habitual dietary patterns and diet quality. Nutrition education was included because nutrition-related knowledge may affect food choices and the ability to maintain diet quality under shift-work conditions. We also considered other potential confounders, including profession, meal skipping, and total energy intake. However, given the sample size and the within-subject design, we aimed to avoid overfitting and also preserve model parsimony by intentionally limiting the number of covariates in the regression models.

For each day condition, predictors were entered in two sequential models, with DQI total score specified as the dependent variable. Model 1 included age, sex, BMI, and nutrition education. In Model 2, condition-specific UPFECP was added to evaluate whether UPF consumption explained additional variance in DQI total score beyond age, sex, BMI, and nutrition education. For the non-shift day analysis, UPFECP calculated from non-shift day food consumption records was entered, whereas for the shift day analysis, UPFECP calculated from shift day food consumption records was entered. Sex was dummy-coded as male = 0 and female = 1, and nutrition education was dummy-coded as no = 0 and yes = 1.

**Fig. 1:**
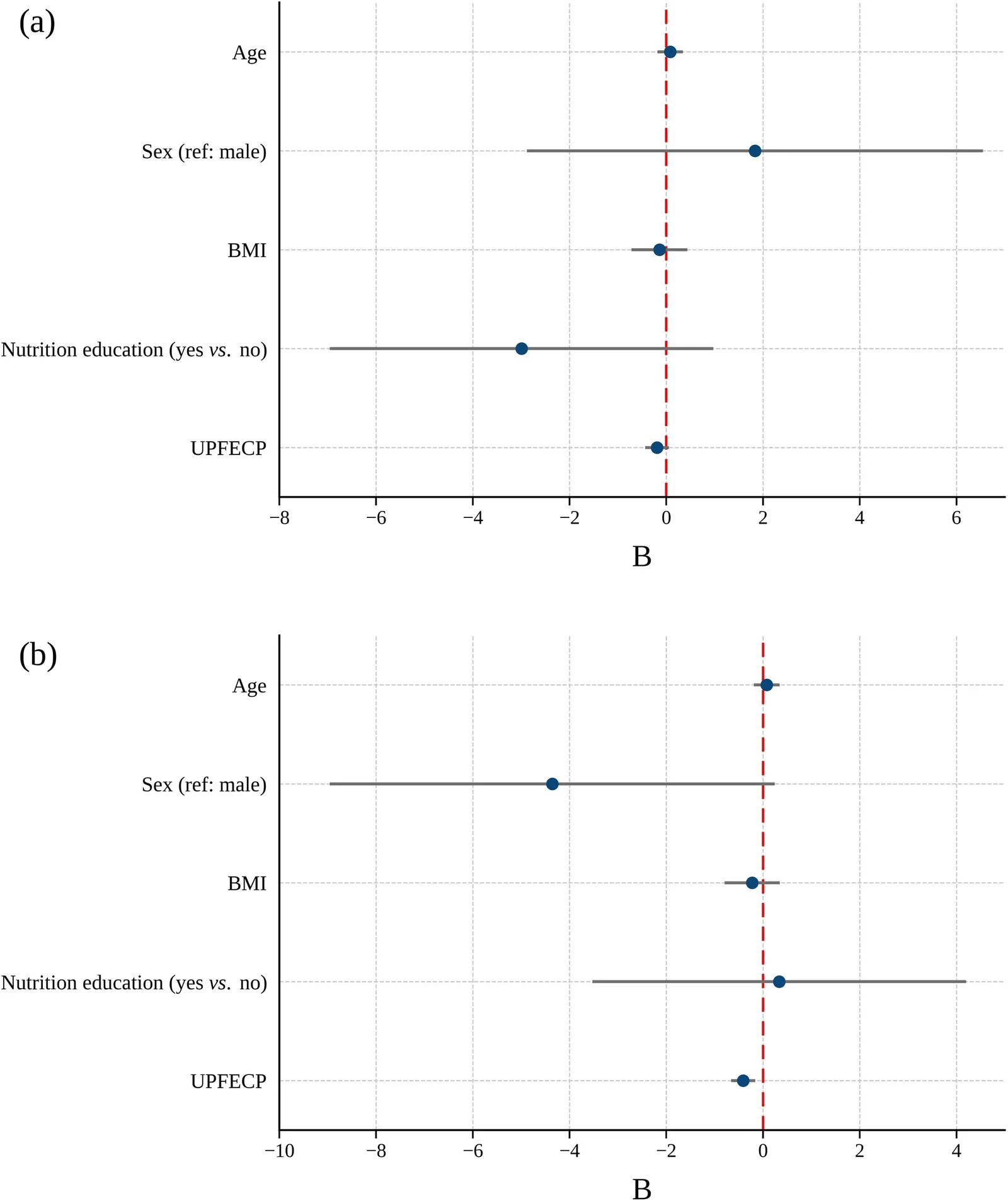
Visual summary of the final adjusted hierarchical linear regression models for non-shift day (**a**) and shift day (**b**). Points indicate unstandardized regression coefficients (B), and horizontal lines indicate 95% confidence intervals. The red dashed vertical line represents B = 0. Sex was coded with male as the reference category, and nutrition education was coded as yes vs. no. Abbreviations: BMI: Body mass index; UPFECP: Ultra-processed food energy contribution percentage

Before model interpretation, regression assumptions were checked. Linearity, normality of residuals, and homoscedasticity were evaluated using residual plots, histograms, and Q–Q plots [18]. Model fit was evaluated using R², adjusted R², the F statistic, and the change in R² (ΔR²) after adding UPFECP. Regression coefficients were reported as unstandardized coefficients (B), standard errors (SE), standardized coefficients (β), 95% confidence intervals (CIs), t values, and p values. Multicollinearity was assessed using tolerance and variance inflation factor (VIF) values, and independence of residuals was evaluated using the Durbin–Watson statistic. Based on recent regression-based health studies, tolerance values above 0.20 [19], VIF values below 5 [20, 21], and Durbin–Watson values close to 2 or within the range of 1.5–2.5 [21] were considered acceptable. A two-sided p value < 0.05 was considered statistically significant. Regression coefficients from the final adjusted models for each day condition were visualized using forest plots (Fig. 1).

## Results

Of the 60 healthcare workers (34.5 ± 7.40 years) participating in the study, 65.0% were male and 51.7% were nurses. Notably, the vast majority of participants reported increased consumption of caffeinated beverages (96.7%), sugary snacks (88.3%), and sugary drinks (70.0%) on shift days. In addition to these changes in dietary intake, the number of main meals decreased significantly on shift days compared to non-shift days (2.7 ± 0.45 vs. 2.6 ± 0.67; *p* = 0.032). Although overall meal skipping did not significantly differ between conditions (*p* = 0.261), lunch was most frequently skipped on shift days (58.3%), while breakfast was more often skipped on non-shift days (61.7%) (*p* < 0.001) (Table 1).

**Table 1:** Socio-demographic characteristics and nutritional habits of participants (*n* = 60)

Variables	*n* (%)		
Socio-demographic Characteristics			
Age (years)^*^	34.5 ± 7.40		
BMI (kg/m^2^)^*^	24.2 ± 4.24		
Gender			
Male	39 (65.0)		
Female	21 (35.0)		
Profession			
Nurse	31 (51.7)		
Doctor	9 (15.0)		
Health technician	20 (33.3)		
Nutrition education			
Yes	42 (70.0)		
No	18 (30.0)		
Consumption Changes (During Shift)			
Caffeinated beverages	58 (96.7) ↑		
Sugary drinks	42 (70.0) ↑		
Sweet/sugary snacks	53 (88.3) ↑		
Salty/packaged snacks	41 (68.3) ↑		

Nutritional Habits	Non-shift day	Shift day	*p*-value
	n (%)	n (%)	
Main meals^*^	2.7 ± 0.45	2.6 ± 0.67	**0.032** ^**¥**^
Snack meals^*^	0.2 ± 0.52	0.3 ± 0.48	0.128^¥^
Meal skipping			0.261^§^
Yes	17 (28.3)	20 (33.3)	
Sometimes	27 (45.0)	28 (46.7)	
Skipped meal			**< 0.001** ^**§**^
Breakfast	37 (61.7)	13 (21.7)	
Lunch	7 (11.7)	35 (58.3)	

^*^$\overline{\mathrm{x}}$±SD. ^¥^Paired samples t-test (*p* < 0.05), ^§^McNemar test (*p* < 0.05). ↑: Increase

Table 2 summarizes participants’ daily energy and nutrient intakes. On shift days, participants had a lower daily energy intake (2624.8 ± 816.53 kcal) than on non-shift days (2826.6 ± 857.77 kcal) (*p* = 0.025). Carbohydrate intake contributed less to total daily energy on shift days (47.6 ± 6.82%) compared to non-shift days (51.9 ± 6.15%), while fat contributed more (37.3 ± 5.98% vs. 33.0 ± 5.43%; *p* < 0.001). Concurrently, specific fatty acid profiles changed significantly; daily intake of saturated fatty acids (SFA) increased from 31.3 ± 13.79 g on non-shift days to 35.3 ± 13.03 g on shift days, whereas polyunsaturated fatty acids (PUFA) decreased from 29.5 ± 10.79 g to 27.1 ± 9.21 g (*p* < 0.001). Dietary fiber intake was also lower on shift days (23.9 ± 7.99 g vs. 26.6 ± 7.55 g; *p* = 0.010). More participants did not meet vitamin B_6_, iron, and zinc requirements on shift days (*p* < 0.05).

**Table 2:** Daily energy and nutrient intake and adequacy status (RDA, %)

Energy and nutrient intake	Non-shift day	Shift day	*p*-value
Energy (kcal)^*^	2826.6 ± 857.77	2624.8 ± 816.53	**0.025** ^**¥**^
Energy adequacy (< RDA, n%)	5 (8.3)	4 (6.7)	0.534^§^
Carbohydrate (E%)^*^	51.9 ± 6.15	47.6 ± 6.82	**< 0.001** ^**¥**^
Protein (E%)^*^	15.1 ± 2.50	15.1 ± 2.27	0.944^¥^
Fat (E%)^*^	33.0 ± 5.43	37.3 ± 5.98	**< 0.001** ^**¥**^
Dietary fiber (g)^*^	26.6 ± 7.55	23.9 ± 7.99	**0.010** ^**¥**^
Saturated fatty acids (g)^*^	31.3 ± 13.79	35.3 ± 13.03	**< 0.001** ^**¥**^
Polyunsaturated fatty acids (g)^*^	29.5 ± 10.79	27.1 ± 9.21	**< 0.001** ^**¥**^
Vitamin C (< RDA, n%)	11 (18.3)	9 (15.0)	0.926^§^
Vitamin B_6_ (< RDA, n%)	4 (6.7)	5 (8.3)	**< 0.001** ^**§**^
Vitamin B_12_ (< RDA, n%)	1 (1.7)	2 (3.3)	0.251^§^
Iron (< RDA, n%)	12 (20.0)	15 (25.0)	**< 0.001** ^**§**^
Zinc (< RDA, n%)	1 (1.7)	2 (3.3)	**0.027** ^**§**^

*Abbreviations: RDA *Recommended Daily Allowance, *E *Energy

^*^$\overline{\mathrm{x}}$±SD, ^¥^Paired samples t-test (*p* < 0.05), ^§^McNemar test (*p* < 0.05)

According to the NOVA classification, the contribution of unprocessed or minimally processed foods to daily energy intake decreased from 48.5% to 42.4% on shift days (*p* < 0.001). Meanwhile, the contribution of processed foods increased from 24.1% to 28.4% (*p* = 0.006). Additionally, the contribution of UPF rose from 8.6% to 11.8% on shift days (*p* = 0.002) (Table 3).

**Table 3:** Daily energy intake according to NOVA food group classification. Paired samples t-test (*p* < 0.05)

NOVA Food Classification	Energy (kcal/day)				*p*-value
	Non-shift day		Shift day		
	$\overline{\mathrm{x}}$±SD	Energy %	$\overline{\mathrm{x}}$±SD	**Energy %**	
Unprocessed/minimally processed	1373.1 ± 488.81	48.5	1094.0 ± 355.54	42.4	**< 0.001**
Processed culinary ingredients	531.6 ± 224.12	18.8	458.1 ± 223.43	17.4	0.154
Processed foods	705.5 ± 396.72	24.1	778.0 ± 457.76	28.4	**0.006**
Ultra-processed foods	228.3 ± 196.51	8.6	294.7 ± 189.84	11.8	**0.002**

Paired samples t-test (p<0.05)

Table 4 shows DQI scores based on the extent to which UPF consumption contributes to daily energy intake. No significant link was found between UPF consumption and DQI total score on non-shift days (*p* = 0.405). On shift days, however, those who consumed the most UPF had significantly lower DQI total scores (57.1 ± 6.98) and DQI adequacy scores (27.2 ± 5.68) than those with low consumption (*p* = 0.003 and *p* < 0.001, respectively).

**Table 4:** Assessment of Diet Quality Index (DQI) scores across tertiles of energy contribution from ultra-processed foods

	Diet Quality Indices	Tertile 1 (Low UPF)	Tertile 2 (Medium UPF)	Tertile 3 (High UPF)	*p*-value
Non-shift day	Cut-off	< 3.6	|3.6–10.1|	> 10.1	
	DQI total score	66.6 ± 6.91	68.5 ± 7.34	65.6 ± 6.62	0.405
	DQI variety	19.1 ± 1.41	19.0 ± 2.05	18.8 ± 1.79	0.862
	DQI adequacy	32.8 ± 5.46	31.3 ± 4.51	31.1 ± 4.81	0.497
	DQI moderation	13.8 ± 6.19	16.4 ± 5.88	14.7 ± 5.67	0.390
	DQI overall balance	0.9 ± 1.77	1.9 ± 2.71	1.0 ± 2.00	0.293
Shift day	Cut-off	< 7.3	|7.3–14.6|	> 14.6	
	DQI total score	63.8 ± 7.89^a^	63.8 ± 5.75^a^	57.1 ± 6.98^b^	**0.003**
	DQI variety	18.5 ± 1.54	19.1 ± 1.71	18.2 ± 1.79	0.237
	DQI adequacy	31.5 ± 3.55^a^	33.0 ± 3.50^a^	27.2 ± 5.68^b^	**< 0.001**
	DQI moderation	12.0 ± 6.23	10.7 ± 5.96	11.0 ± 3.12	0.700
	DQI overall balance	1.8 ± 2.24	1.1 ± 1.52	0.8 ± 1.20	0.174

Compared across tertiles using one-way ANOVA with Bonferroni post-hoc adjustment

*Abbreviation: DQI *Diet Quality Index

^a, b^Values on the same row not sharing the same superscript letters were significantly different (*p* < 0.05)

Hierarchical linear regression analyses were conducted separately for non-shift and shift days to examine whether the condition-specific UPFECP was associated with DQI total score after adjustment for age, sex, BMI, and nutrition education. Detailed numerical regression estimates, including B coefficients, SE, standardized β coefficients, 95% CIs, model fit indices, and diagnostic statistics, are presented in Table 5. Figure 1 provides a simplified visual summary of the final adjusted models, showing only the unstandardized coefficients and their 95% CIs to facilitate interpretation of the direction, magnitude and precision of the associations.

**Table 5:** Hierarchical linear regression analyses predicting Diet Quality Index total score non-shift and shift days

**Outcome / Predictor**		**Model 1**					**Model 2**					**Model 2 95% CI for B**		**Tolerance Range**	**VIF Range**
**Non-shift day DQI total score**		**B**	**SE**	**β**	**t**	**p**	**B**	**SE**	**β**	**t**	**p**	**Lower**	**Upper**		
Constant		66.434	7.722	-	8.603	<0.001	70.582	8.065	-	8.752	<0.001	54.413	86.751	Model 1:0.552 to 0.979	Model 1:1.021 to 1.812
Age		0.109	0.131	0.116	0.829	0.411	0.081	0.131	0.086	0.621	0.537	-0.181	0.344		
Sex (ref: male)		1.647	2.379	0.114	0.692	0.492	1.835	2.351	0.127	0.780	0.439	-2.879	6.548		
BMI		-0.058	0.287	-0.035	-0.201	0.841	-0.141	0.288	-0.086	-0.489	0.627	-0.718	0.437	Model 2:0.533 to 0.951	Model 2:1.052 to 1.875
Nutrition education(yes*vs.*no)		-3.520	1.974	-0.234	-1.783	0.080	-2.992	1.977	-0.199	-1.513	0.136	-6.956	0.972		
UPFECP		-	-	-	-	-	-0.192	0.122	-0.219	-1.573	0.122	-0.436	0.053		

Shift day DQI total score		B	SE	β	t	p	B	SE	β	t	p	Model 2 95% CI for B		Tolerance Range	VIF Range
												Lower	Upper		
Constant		56.888	8.085	-	7.036	<0.001	70.520	8.511	-	8.286	<0.001	53.456	87.584	Model 1:0.552 to 0.979	Model 1:1.021 to 1.812
Age		0.200	0.138	0.197	1.456	0.151	0.077	0.132	0.075	0.580	0.564	-0.188	0.341		
Sex (ref: male)		-4.199	2.491	-0.268	-1.685	0.098	-4.357	2.294	-0.279	-1.899	0.063	-8.956	0.242		
BMI		-0.011	0.300	-0.006	-0.037	0.971	-0.224	0.284	-0.126	-0.788	0.434	-0.793	0.345	Model 2:0.524 to 0.955	Model 2:1.047 to 1.910
Nutrition education(yes*vs.*no)		-0.666	2.067	-0.041	-0.322	0.748	0.335	1.927	0.021	0.174	0.863	-3.528	4.197		
UPFECP		-	-	-	-	-	-0.411	0.124	-0.436	-3.302	**0.002**	-0.660	-0.161		

Outcome	Model	Predictors	R²	Adjusted R²			ΔR²	F	p			F change	p for ΔR²	Durbin-Watson	
Non-shift day DQI total score	Model 1	Age, sex, BMI, nutrition education	0.073	0.005			-	1.080	0.375			-	-	-	
Non-shift day DQI total score	Model 2	Model 1 predictors + UPFECP_NS	0.113	0.031			0.041	1.382	0.246			2.473	0.122	1.688	
Shift day DQI total score	Model 1	Age, sex, BMI, nutrition education	0.132	0.068			-	2.084	0.095			-	-	-	
Shift day DQI total score	Model 2	Model 1 predictors + UPFECP_S	0.277	0.211			0.146	4.147	**0.003**			10.900	0.002	2.037	

Model 1 included age, sex, BMI, and nutrition education. Model 2 included the same covariates plus condition-specific UPFECP. Sex was dummy-coded as male = 0 and female = 1. Nutrition education was dummy-coded as no = 0 and yes = 1. Tolerance and VIF ranges are presented for each model where applicable. ΔR² refers to the additional explained variance after adding UPFECP to the covariate-adjusted model. The Durbin-Watson statistic was evaluated in the final model for each condition

*Abbreviations: BMI *Body mass index, *CI *Confidence interval, *DQI *Diet Quality Index, *SE *Standard error, *UPFECP *Ultra-processed food energy contribution percentage, *UPFECP_NS *UPFECP on non-shift days, *UPFECP_S *UPFECP on shift days, *VIF *Variance inflation factor

On non-shift days, the covariate-adjusted model including age, sex, BMI, and nutrition education explained 7.3% of the variance in DQI total score; however, the model was not statistically significant (R²=0.073, adjusted R²=0.005, F = 1.080, *p* = 0.375). In this model, none of the covariates were significantly associated with DQI total score. When non-shift day UPFECP was added to the model, the explained variance increased to 11.3%, but this increase was not statistically significant (ΔR²=0.041, F change = 2.473, *p* = 0.122). In the final non-shift day model, UPFECP was inversely but not significantly associated with DQI total score (B= -0.192, SE = 0.122, β= -0.219, 95% CI: -0.436 to 0.053, t= -1.573, *p* = 0.122). These findings indicate that, after adjustment for relevant covariates, the percentage of daily energy intake derived from UPF was not a significant predictor of diet quality on non-shift days.

On shift days, the covariate-adjusted model explained 13.2% of the variance in DQI total score and approached, but did not reach, statistical significance (R²=0.132, adjusted R²=0.068, F = 2.084, *p* = 0.095). In this model, none of the covariates were significantly associated with DQI total score. After shift day UPFECP was added, the explained variance increased to 27.7%, and the final model was statistically significant (R²=0.277, adjusted R²=0.211, F = 4.147, *p* = 0.003). The addition of UPFECP significantly improved model fit (ΔR²=0.146, F change = 10.900, *p* = 0.002). In the final shift day model, UPFECP was inversely associated with DQI total score after adjustment for age, sex, BMI, and nutrition education (B= -0.411, SE = 0.124, β= -0.436, 95% CI: -0.660 to -0.161, t= -3.302, *p* = 0.002). In practical terms, each 1-percentage-point increase in the proportion of daily energy derived from UPF on shift days was associated with an approximately 0.41-point lower DQI total score. This suggests that a higher dietary share of UPF energy contribution during shift days may be associated with poorer overall diet quality. In contrast, this association was not statistically significant on non-shift days.

Diagnostic indices indicated no problematic multicollinearity in either final model, with acceptable tolerance and VIF values. Durbin–Watson statistics were 1.688 for the non-shift day model and 2.037 for the shift day model, supporting independence of residuals.

## Discussion

This study used a within-subject design to show the hidden impact of shift work on healthcare workers’ eating habits and diet. Unlike cross-sectional studies, our approach reduces individual differences and better detects shift-related dietary changes. On shift days, total daily energy intake decreased. However, the proportion of UPF in the diet increased. Dietary behaviors changed significantly, leading to a shift toward a lower-quality dietary pattern. Crucially, the two most pivotal findings of this investigation are the increase in UPF consumption during shift days and its independent, robust negative association with overall diet quality, which persists even after controlling for baseline individual characteristics.

Shift work substantially impacts the dietary habits and nutritional quality of healthcare workers. The vast majority of participants reported increased consumption of caffeinated beverages (96.7%) and sugary snacks (88.3%) during their shifts. This finding aligns with the literature, which indicates that night-shift workers turn to carbohydrates and stimulants to quickly boost energy and maintain alertness while coping with fatigue [22, 23]. Shift work disrupts the circadian rhythm, which in turn alters both food preferences and meal patterns. Notably, a recent systematic review and meta-analysis demonstrated that shift workers exhibit irregular meal patterns, which are associated with metabolic risk [7]. Moreover, while some studies report a decrease in daily energy intake on shift days [23], others indicate daily energy intake is similar on shift and non-shift days [24, 25]. The observed decrease in total daily energy intake on shift days was primarily driven by a lower number of main meals and frequent lunch skipping (58.3%). Crucially, this reduction in daily energy intake should not be misinterpreted as intentional portion control or a favorable caloric restriction. Instead, it seems the energy shortfall from skipped main meals is compensated by energy-dense, high-fat convenience options, nutritionally poor ultra-processed snacks and sugary or caffeinated beverages—foods readily accessible in the hospital setting. Shift work scheduling among healthcare workers alters dietary patterns by reducing total daily energy intake but compromising diet quality. This leads to micronutrient deficiencies and increased consumption of UPF. As a result, differences in macronutrient distribution emerge on shift days; carbohydrates contribute less, and fats contribute more to daily energy intake during these periods. Importantly, our results expand on this shift by demonstrating a significant increase in absolute SFA intake alongside a concomitant reduction in PUFA intake during shift days. This unfavorable change in fat quality likely reflects the increased reliance on convenience snacks and ultra-processed options typically available during night duties, which often feature high amounts of saturated fats at the expense of essential fatty acids. Additionally, decreased daily intake of iron, vitamin B_6_, and zinc—nutrients important in the synthesis of neurotransmitters that regulate the sleep-wake cycle [26]—may cause post-shift chronic fatigue, mental burnout, and reduced cognitive performance among healthcare workers.

Our study highlights marked differences in dietary patterns between shift work and non-shift work days, particularly an increased consumption of UPF during shift work days. This difference may be partly driven by cortisol dynamics, which alter leptin sensitivity and insulin resistance late at night. These hormonal shifts prompt individuals to select highly palatable, energy-dense foods to cope with night-shift fatigue [27–29]. Additionally, the nighttime hospital environment restricts access to healthy food, forcing reliance on vending machines that predominantly stock nutritionally poor, highly processed options [9]. The significant decrease in daily energy intake from unprocessed/minimally processed foods on shift days shows that shift work—a necessity for healthcare workers—affects diet quality. Since high UPF consumption increases the risk of long-term diseases [30], institutions should ensure that unprocessed/minimally processed food options and healthy meals are readily available to healthcare workers during night shifts to safeguard their metabolic health.

To further elucidate the independent contribution of UPF consumption to diet quality, hierarchical linear regression models were constructed. The regression findings provide a practical interpretation of the relationship between UPF consumption and diet quality during shift work. A key finding of this study is that, after adjusting for major confounding factors (age, sex, BMI, prior nutrition education), the UPFECP remained a strong and significant negative predictor of DQI total scores during shift days (β= -0.436, *p* = 0.002). Interestingly, this independent negative association was not observed on non-shift days. This discrepancy highlights that the detrimental impact of UPF on overall diet quality is not merely a reflection of baseline individual characteristics or static dietary habits, but is acutely exacerbated by the environmental, logistical, and physiological challenges unique to the shift-work window [7]. This independent relationship aligns with emerging evidence suggesting that the interplay between circadian disruption and food environments creates a highly vulnerable state for dietary degradation [31]. While traditional cross-sectional studies often confound shift-work diets with socioeconomic status or pre-existing metabolic profiles, our within-subject regression approach isolates UPF intake as a direct driver of diminished diet quality. The fact that the model accounted for a significant portion of the variance on shift days underscores that institutional food environments—such as the overavailability of ultra-processed snacks in hospital vending machines during night shifts—act as critical leverage points [9]. Therefore, interventions aiming to improve healthcare workers’ nutritional status must go beyond general nutrition education, which was not a significant predictor in our models, and directly target the structural reduction of UPF availability in workplace settings.

This study has several notable strengths. Foremost is its within-subject design, which rigorously compares individuals across shift and non-shift days. Using each participant as their own control eliminates time-invariant confounding factors and genetic variability, enhancing internal validity. However, some limitations should be acknowledged. Although 24-hour recalls are widely utilized in nutritional epidemiology, they remain vulnerable to recall and social desirability biases. Additionally, while dietary recalls were conducted face-to-face by a trained dietitian to minimize error, the lower total energy intake reported during shift days might partially reflect potential underreporting of nocturnal snacks due to shift-related fatigue or cognitive overload. Furthermore, a small convenience sample was recruited from a single medical center in Türkiye, which limits representativeness and generalizability. Finally, no objective biomarkers of dietary intake, circadian disruption, cortisol, or metabolic health were collected, so the proposed mechanisms could not be tested.

## Conclusion

This within-subject study demonstrates that shift work among healthcare professionals is associated with increased UPF consumption and poorer diet quality. These findings highlight the need for institutional strategies to improve access to healthy, minimally processed foods during night shifts.

## Supplementary Information

Supplementary Material 1.

## Data Availability

Research data are available from the corresponding author upon reasonable request.

## Abbreviations

ANOVA: One-way analysis of variance
BMI: Body mass index
CI: Confidence interval
DQI: Diet Quality Index
EFSA: European Food Safety Authority
FAO: Food and Agriculture Organization
PUFA: Polyunsaturated fatty acids
SD: Standard deviation
SE: Standard errors
SFA: Saturated fatty acids
UPF: Ultra-processed foods
UPFECP: Ultra-processed foods energy contribution percentage
UPFECP_NS: UPFECP on non-shift days
UPFECP_S: UPFECP on shift days
VIF: Variance inflation factor
