# Supplementary material for: Ultra-processed foods consumption and diet quality during shift work among healthcare workers: a within-subject design
Source: BMC Public Health. 2026 Jul 29;26:3062. doi: 10.1186/s12889-026-28697-z (PMC13700257; doi:10.1186/s12889-026-28697-z)
Supplement: Supplementary file 1 [file 12889_2026_28697_MOESM1_ESM.docx]

**Supplementary File: Survey Questionnaire**

**Title:** A survey study to assess the consumption of ultra-processed foods and diet quality among healthcare workers on both shift and non-shift workdays

Survey number:	Date:

A. **General Information**
1- Age: ……… (year)
2- Body weight: ….. (kg)	Height: ……… (cm)	BMI: ………. (kg/m^2^)
3- Gender: 1. Male	2. Female
4- Profession: 1. Nurse	2. Doctor	3. Health Technician
5- Have you ever taken a nutrition education?	1. Yes	2. No
6- How many meals do you eat a day?

Non-shift work day: a. ………………… main meal	b. ………………… snack meal

Shift work day : a. ………………… main meal	b. ………………… snack meal

7- Do you skip main meals? (If your answer is no, you can skip to question 9)

Non-shift work day: 1. No	2. Yes	3. Sometimes

Shift work day:	1. No	2. Yes	3. Sometimes

8- Which main meal do you skip most often?

Non-shift work day: 1. Breakfast	2. Lunch	3. Dinner

Shift work day:	1. Breakfast	2. Lunch	3. Dinner

9- Indicate any changes in your consumption of the foods/beverages listed below on a shift work day. (Check the box)

|  | **Changes in consumption on a shift work day** | | |
| --- | --- | --- | --- |
|  | It doesn't change | Increasing | Decreasing |
| Caffeinated beverages |  |  |  |
| Sugary drinks |  |  |  |
| Sweet/sugary snacks |  |  |  |
| Salty/packaged snacks |  |  |  |

B. **24-HOUR FOOD CONSUMPTION RECORD- Non-shift work day-1**

| **Hour** | **Description of food or drink** | **Contents** | **Measurement** | **Amount consumed** |
| --- | --- | --- | --- | --- |
|  |  |  |  |  |
|  |  |  |  |  |
|  |  |  |  |  |
|  |  |  |  |  |
|  |  |  |  |  |

**B.	24-HOUR FOOD CONSUMPTION RECORD- Non-shift work day-2**

| **Hour** | **Description of food or drink** | **Contents** | **Measurement** | **Amount consumed** |
| --- | --- | --- | --- | --- |
|  |  |  |  |  |
|  |  |  |  |  |
|  |  |  |  |  |
|  |  |  |  |  |
|  |  |  |  |  |

**B.	24-HOUR FOOD CONSUMPTION RECORD- Shift work day-1**

| **Hour** | **Description of food or drink** | **Contents** | **Measurement** | **Amount consumed** |
| --- | --- | --- | --- | --- |
|  |  |  |  |  |
|  |  |  |  |  |
|  |  |  |  |  |
|  |  |  |  |  |
|  |  |  |  |  |

**B.	24-HOUR FOOD CONSUMPTION RECORD- Shift work day-2**

| **Hour** | **Description of food or drink** | **Contents** | **Measurement** | **Amount consumed** |
| --- | --- | --- | --- | --- |
|  |  |  |  |  |
|  |  |  |  |  |
|  |  |  |  |  |
|  |  |  |  |  |
|  |  |  |  |  |
